# Establishment and Validation of a Novel Microgravity Simulation Platform for Ground-Based Animal Experiments

**DOI:** 10.3390/ijms27198806

**Published:** 2026-10-01

**Authors:** Chetali Gurung, Junfeng Li, Bin Teng, Dong Liang, Jian V. Zhang, Pei-Gen Ren

**Affiliations:** 1 Center for Energy Metabolism and Reproduction, Institute of Biomedicine and Biotechnology, Shenzhen Institutes of Advanced Technology, Chinese Academy of Sciences, Shenzhen 518055, China; gurung@siat.ac.cn (C.G.); lijf@siat.ac.cn (J.L.);; 2 Shenzhen College of Advanced Technology, University of Chinese Academy of Sciences, Beijing 100049, China; 3 Research Center for Cancer Immunology, Institute of Biomedicine and Biotechnology, Shenzhen Institutes of Advanced Technology, Chinese Academy of Sciences, Shenzhen 518055, China

**Keywords:** simulated microgravity, inflammation, muscle atrophy, muscle fiber shift, disuse osteoporosis

## Abstract

Ground-based analog models, such as the hindlimb unloading (HU) technique, have been used for several decades in simulating microgravity effects on musculoskeletal studies. However, this model has seen limited technological advancement in several aspects. Here, the design and validation of the Microgravity Simulated Platform (MSP), a refined evolution of the classic HU model, is presented. The multipurpose engineered MSP apparatus is designed to overcome key limitations of existing models by enabling simultaneous study of a larger number of mice in compact space, with fewer lesions, less inflammation, and central axis rotation control that offers potential for future hypergravity studies. This study presents a comparative analysis with the HU and normal WT (NOR) models and demonstrates that the MSP recapitulates the classic physiological markers of disuse observed during spaceflight, particularly in the musculoskeletal system. Critically, the results indicate that the MSP not only provides a comparable simulation of microgravity-induced effects but also offers enhancements in experimental consistency and animal well-being. In conclusion, the MSP represents a significant advancement in ground-based space life sciences research, providing a more refined, efficient, high-throughput, and extensible platform for elucidating the pathophysiological mechanisms of mechanical unloading and for testing prospective countermeasures.

## 1. Introduction

Exposure to reduced gravity, ionizing radiation, and other environmental factors during long-term spaceflight poses substantial health challenges for astronauts, cosmonauts, and taikonauts [1]. Many key physiological changes, including reduced bone integrity; decreased muscle mass; altered intervertebral discs; modifications to biomechanical and endocrine metabolism; cephalic fluid shifts; and straightening and stiffening of the spine, which may result in backache, are all consequences of experiencing reduced gravity on the muscles and bones [2,3,4,5]: muscles undergo atrophy, while bones experience deconditioning, increasing the risk of fractures and disuse osteoporosis [6,7]. Previous studies demonstrated that the soleus muscle undergoes up to 30% mass reduction following three weeks in space [8]. Additionally, bone mineral density decreases by approximately 2% over the same period, reflecting the adverse effects of mechanical unloading. As a result, overall body mass declines by approximately 2.4% over a three-week duration in microgravity [9]. These effects compromise the viability of extended missions and impair astronauts’ mobility upon their return to Earth. Recently, multidimensional analyses from the NASA Twins Study revealed that the astronaut twin experienced a significant physiological impact during prolonged spaceflight, including a 7% reduction in body mass over the course of one year aboard the International Space Station [10].

Although small animal models are commonly used to study the physiological effects associated with spaceflight, several challenges persist, including limited flight opportunities, restrictions on the number of animals that can be flown at a time, and the complexity of onboard experimental setups [11]. Our colleagues have recently completed the first test of mice living in the China Manned Space Agency (CMSA) and returning to Earth, highlighting the importance of laboratory rodents in space biomedical research and the experimental limitations in space [12]. To address the limit on the number of flown animals, ground-based analogs have been developed. To our knowledge, Morey et al. was the first to introduce the hindlimb unloading (HU) model in rats, in which the animals are suspended by their tails, which elevates the hindlimbs and simulates the cephalic fluid shift observed in microgravity [13]. Since its development, the hindlimb unloading model has been widely used for investigating microgravity-induced physiological changes, particularly in the musculoskeletal and cardiovascular systems [14]. However, the HU model has been associated with significant increased chronic inflammation and tissue necrosis where surgical tape or other tools are applied. Since then, attempts have been made to improve the method for over a decade now, with multiple modifications such as tail surgery, but still, certain complications restrict broader application and may result in animal distress and decline in overall health, as well as introduce uncertainty into studies sensitive to chronic inflammatory conditions, such as widely recognized research on metabolic diseases [15]. Consequently, to resolve this challenge, we propose an innovative and refined approach to simulate microgravity at the ground level while minimizing animal discomfort, inflammation, and necrosis, thereby enhancing the reliability and reproducibility of the experimental outcomes and improving animal treatment.

The novel ground-based simulation platform Microgravity Simulated Platform (MSP) is versatile equipment with multiple integrated functions that extensively support controlled research. Some of the key features include a high-throughput ergonomic design facilitating larger groups of subjects, sample collection with detachable trays for feces and urine collection, and controlled axis rotation for future hypergravity studies. Moreover, the platform is equipped with integrated thermal regulation via a heating pad system to maintain a stable ambient temperature throughout the experiment. Each mouse is provided with an individual compartment containing food and water, strategically positioned within easy reach to ensure uninterrupted access and normal feeding behavior. We are also the first to develop a mouse jacket for unloading, which covers a large surface area of the mouse body to provide improved stability and cause significantly less distress to the animals. Our findings revealed that improving the comfort and unloaded surface area for animal subjects in our ground-based apparatus resulted in superior control over inflammation and yielded more reliable physiological results compared with the HU model. Hence, this article focuses on establishing an innovative ground-based platform for simulating microgravity and highlights its potential for more humane and physiologically relevant studies compared with tail suspension methods.

## 2. Results

### 2.1. Design and Validation of the MSP Model to Simulate Microgravity at the Ground Level

**Figure 1:**
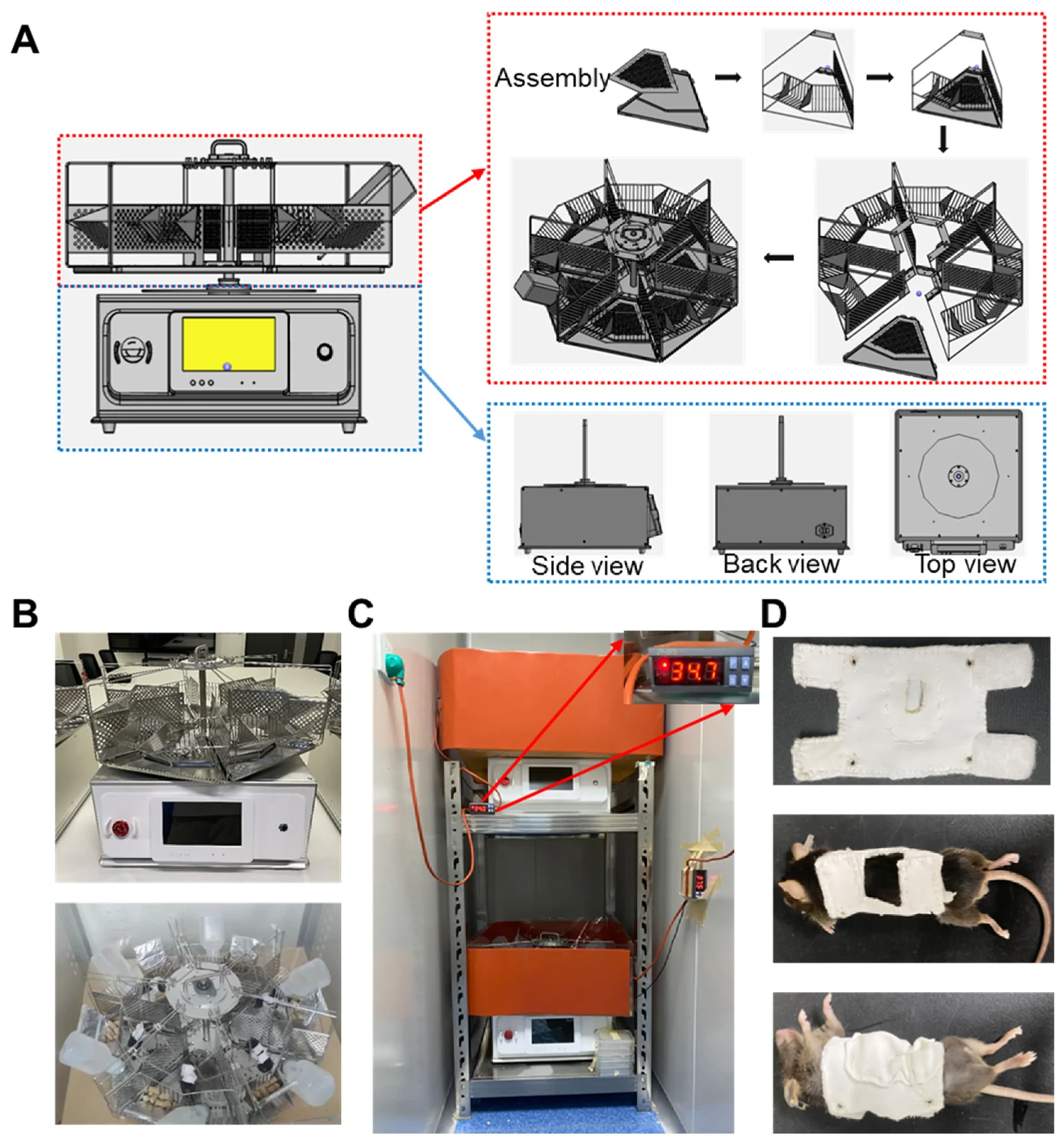
Design, prototype, and accessories of the MSP system. (**A**) Prototype assembly of each component of the platform indicated in the upper panel and lower rotor machine from different views. (**B**) Complete configuration of the machine without animals (upper image) and top view of the machine containing mice in an unloaded state (lower image). (**C**) Arrangement of the working MSP apparatus in the SPF-animal facility area. (**D**) Animal jacket and demonstration of a mouse wearing the jacket.

With a philosophy that prioritizes ergonomic efficiency, species adaptability, and experimental throughput, we designed a robust, advanced multifunctional simulation platform to overcome the major shortcomings in current ground-based models. Following iterative prototyping, we selected a hexagonal configuration as the optimal pattern, equally partitioning the apparatus into six independent, radially assembled compartments. Each space is capable of housing an experimental animal, a configuration that enhances spatial efficiency while ensuring physical isolation to eliminate any social stressors. Another novelty of this apparatus is its capacity for controlled rotation about the central axis rotation system, providing the mechanical capacity to generate hypergravity-like conditions, which could be used in future studies to simulate physiological loads associated with spacecraft maneuvers, atmospheric re-entry and landing, or in-flight exercise countermeasures (Figure 1A) (Supplementary Video S1). To facilitate a comprehensive longitudinal study, every unit was equipped with food trays and water access points for continuous monitoring of consumption behavior and to decrease mixing of food and mouse feces, which is commonly observed in the HU model. Moreover, this facilitates the non-disturbance, non-invasive sampling of feces, thus allowing study of the gut microbiota composition and the promotion of cage hygiene, which is not available in the HU model. Animals housed in the MSP apparatus were allowed normal, unrestricted access to food and water, consuming both ad libitum without hesitation, and demonstrated no observable behavioral indicators of stress or physical restriction (Supplementary Video S2). A thermal system apparatus allows heat to be precisely managed for a comfortable 24–26 °C environment to be maintained, therefore avoiding severe drops in ambient temperature, as observed in the HU model, which would produce unreliable experimental results (Figure 1B,C). For comprehensive behavioral monitoring, the MSP also incorporates a camera setup. This integrated camera facilitates continuous surveillance on each animal and records the activity pattern of animals, which could be extended for cognition and learning studies under simulated microgravity.

All unloading procedures, including the transfer of mice using a low-stress jacket system, were well tolerated (Figure 1D). A key component of the wearable jacket is a thoracic area covering a cloth pocket designed for optimal sensor–skin contact. The pocket securely positions the sensor to measure cardiopulmonary pulse, facilitating robust in vivo monitoring of physiological signals, and the measurements could be recorded through LoRa (Long-Range Radio) onto the system. This feature is still under development but will soon be incorporated into the next advanced MSP 2.0 model (Supplementary Figure S1).

For microgravity simulation, the MSP apparatus is configured to maintain a head-down tilt of 30°, with the hindlimbs elevated to achieve complete unloading. The jacket distributes the suspension load across the pelvic region rather than concentrating it on the tail, thereby eliminating the ischemia and necrosis commonly observed in the HU model.

### 2.2. MSP Model, a Sustainable System with Reduced Systemic Inflammation and Prolonged Survival

**Figure 2:**
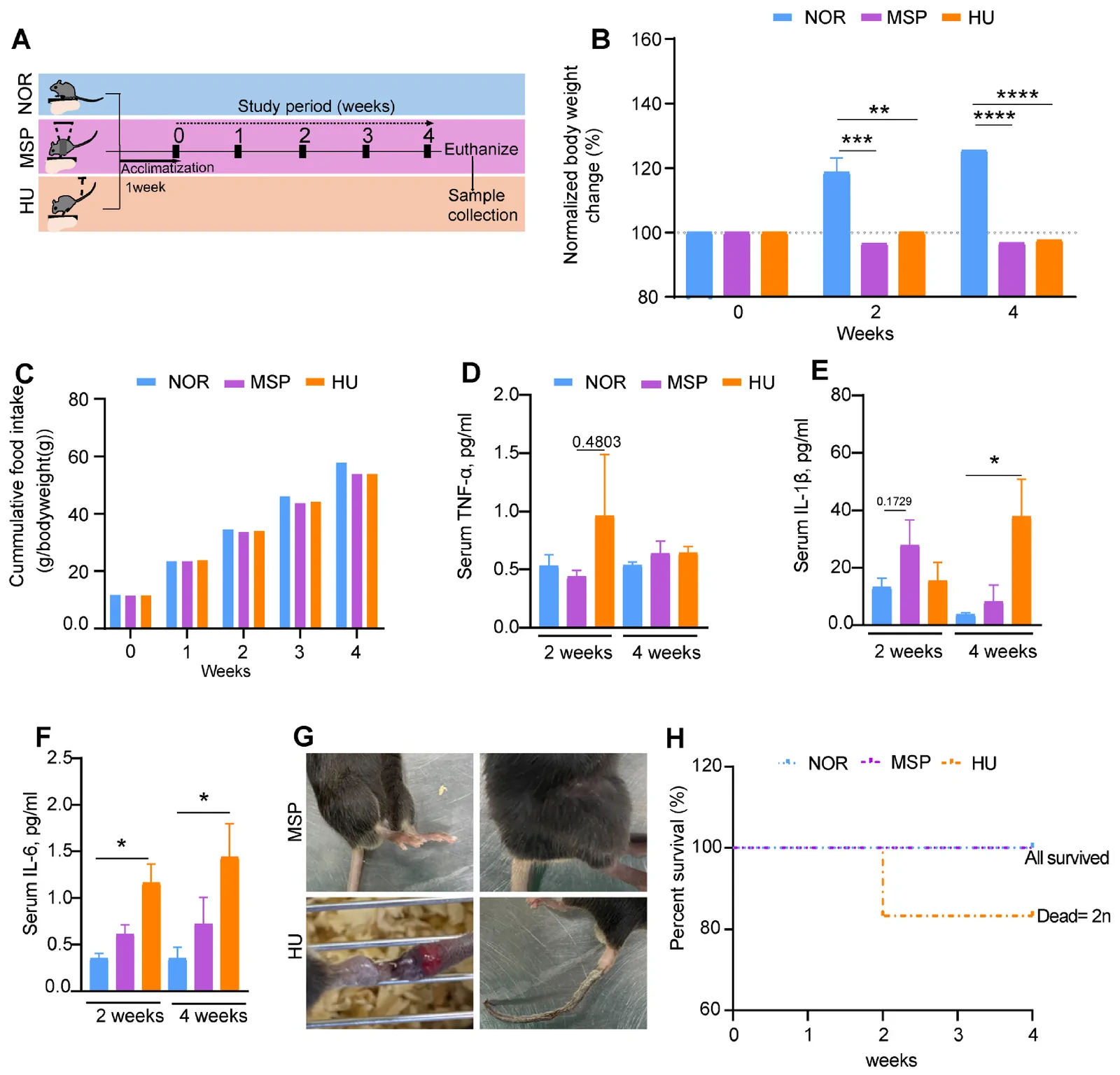
Physiological parameters, inflammatory levels, and survival rate of mice over four weeks on two simulated microgravity systems. (**A**) Timeline of the experiment with three different groups. (**B**,**C**) Body weight and food intake of the NOR (*n* = 9), MSP (*n* = 12), and HU (*n* = 12) groups during four weeks of mechanical unloading. (Cohen’s d: NOR vs. MSP (2 w) = 2.09, NOR vs. HU (2 w) = 1.42, NOR vs. MSP (4 w) = 2.52, NOR vs. HU (4 w) = 2.12.) (**D**–**F**) Inflammatory responses throughout two and four weeks of study, serum TNFα, serum IL-1β, and serum IL-6, respectively (*n* = 4) (Cohen’s d: serum IL-1β—NOR vs. HU (4 w) = −2.04; IL-6—NOR vs. HU (2 w) = −3.22, NOR vs. HU (4 w) = −2.07). (**G**) Representative image of injuries observed in the HU and MSP models. (**H**) Survival curve of the three different groups. Values are means ± SEM. Statistical analysis was performed using one-way ANOVA followed by Tukey’s post hoc test. The results of the one-way analysis of variance are displayed. * *p* < 0.05, ** *p* < 0.01, *** *p* < 0.001, and **** *p* < 0.0001 between groups.

The widely used HU model, while valuable, exhibits several shortcomings, including significant animal distress; chronic pain, linked to the different unloading techniques such as caudal hanging; surgical tail rings; and logistical challenges in dealing with multiple subjects for long-term studies. To assess the efficacy of our newly designed simulation platform, we conducted a direct comparative analysis with the widely used HU model and incorporated normal WT mice (NOR) for a baseline study (Figure 2A). This study aimed to evaluate the extent to which our apparatus mitigates these inherent limitations. A significant reduction in body weight was evident in the MSP and HU groups after two weeks of unloading, which culminated in a maximal decrease the HU and MSP mice compared with in the NOR group following four weeks, as set up in Figure 2B. At four weeks, there was no significant difference in MSP and HU body weight. Decreased food intake was observed in the MSP and HU groups; however, the difference was not significant between the three groups (Figure 2C). The HU model exhibited an elevation in systemic inflammation at three days post unloading, followed by a more pronounced increase at day seven. The levels of pro-inflammatory markers such as serum IL-1β and IL-6 were significantly high in the HU model when compared with the NOR and MSP models; however, no significant difference was found in the serum TNFα between groups (Figure 2D–F). This implies that caudal region unloading triggers a progressive, acute whole-body inflammatory response. Conversely, the MSP-unloaded group remained highly stable and less inflamed throughout this study, suggesting that it provides less distress to the mice. Furthermore, survival was significantly compromised in the HU model, where two mice died during the second week of unloading. These deaths were attributed primarily to signs of hypothermia and an inability to access water, as prolonged hanging caused a decline in mobility, further decreasing the mice’s capacity to regulate optimum body temperature and thus leading to mortality. Similar reports have already been mentioned in previous studies [11]. This outcome correlated with profound swelling necrosis at the unloaded site, which also made the tail vein inaccessible for intravenous administration in future work. In contrast, the MSP model survived throughout the study period and with lower inflammation around the unloading region (Figure 2G,H). Hence, the MSP model provides an ethically and methodologically superior platform for studying microgravity simulation. Its larger body surface unloading mechanism eliminates the systemic stress and mechanical pressure at small regions and provides homogenous distribution of pressure across the whole body. Furthermore, the MSP model allows essential tail vein interventions in the unloaded state, thereby enhancing scientific utility and adherence to the principle of microgravity simulation. Although we have recently extended our validation to include female mice over an eight-week unloading protocol, further investigations comparing MSP findings with real spaceflight data and across different age groups are needed to fully establish the general applicability and translational relevance of the platform (Supplementary Figure S2).

### 2.3. Muscle Fiber Size Reduction and Switches from the Slow to Fast Types in HU and MSP Unloaded Muscle

**Figure 3:**
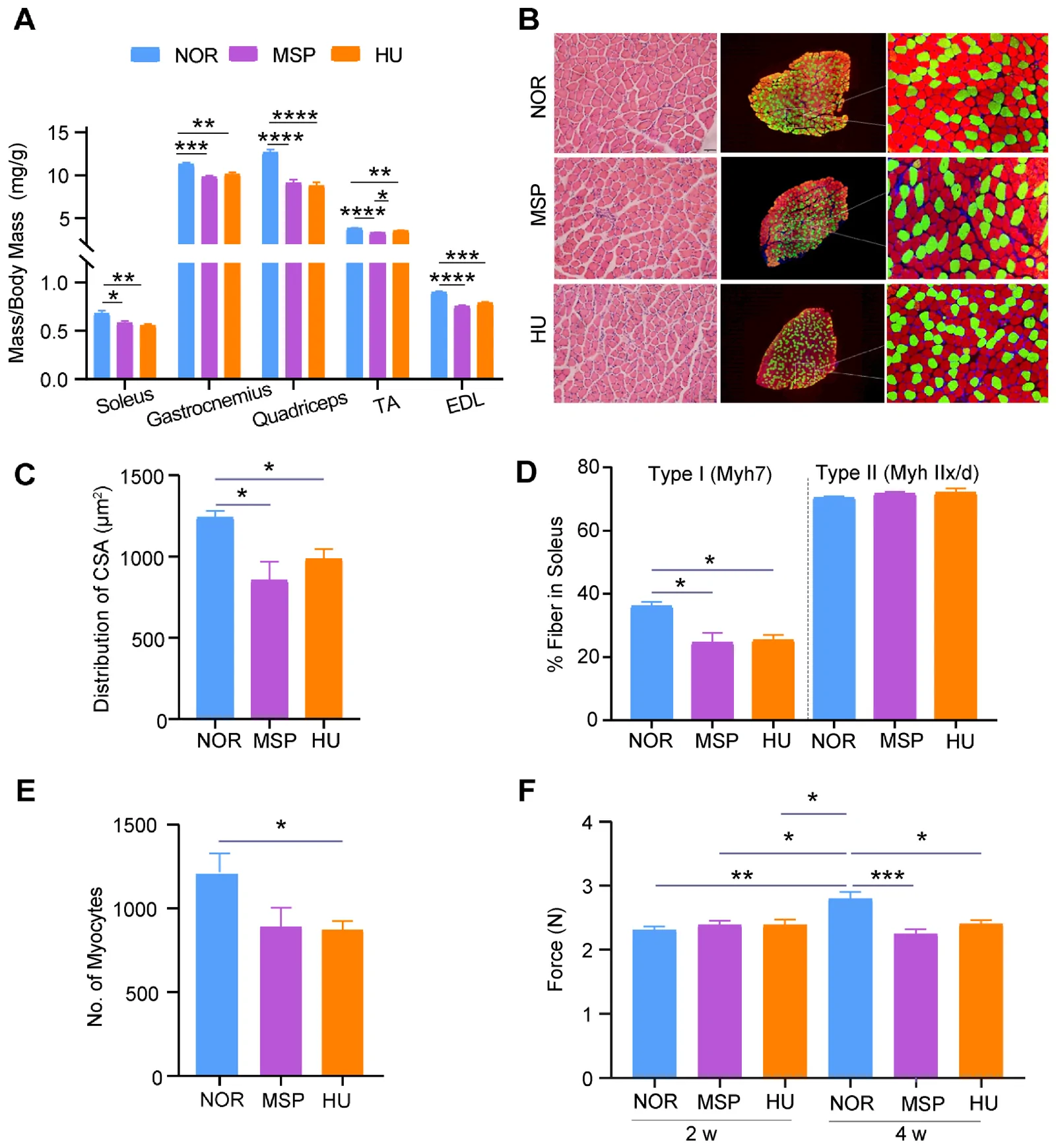
Mechanical unloading in the HU and MSP groups causes muscle mass atrophy, fiber type shift, and muscle weakening. (**A**) Total wet muscle mass of the soleus, gastrocnemius, quadriceps, TA, and EDL by body weight in mg/g at 4 weeks of unloading (*n* = 9–12). (Cohen’s d: soleus—NOR vs. MSP = 1.17, NOR vs. HU = 1.50; gastrocnemius—NOR vs. MSP = 2.50, NOR vs. HU = 1.69; quadriceps—NOR vs. MSP = 2.76, NOR vs. HU = 3.31; TA—NOR vs. MSP = 3.08, NOR vs. HU = 1.14, MSP vs. HU = −1.31; EDL—NOR vs. MSP = 2.59, NOR vs. HU = 1.85.) (**B**) H&E and immunostaining of type I and II muscle fibers of the soleus after 4 weeks of unloading in the NOR, HU, and MSP groups, respectively. Scale bar: 50 µm and 200 µm. (**C**–**E**) Quantification of muscle cross-sectional area and number of myocytes in the soleus, followed by quantification of slow and fast twitch muscle fiber percent between models. (Cohen’s d: distribution of CSA—NOR vs. MSP = 2.56, NOR vs. HU = 2.94; type 1 fiber percent—NOR vs. MSP= 2.72, NOR vs. HU = 3.99; no. of myocytes—NOR vs. HU = 2.33.) (**F**) Grip strength test using four limbs at two and four weeks (*n* = 9–12). (Cohen’s d: NOR (2 w) vs. NOR (4 w) = −1.62, MSP (2 w) vs. NOR (4 w) = −1.13, HU (2 w) vs. NOR (4 w) = −1.18, NOR (4 w) vs. MSP (4 w) = −0.79, NOR (4 w) vs. HU (4 w) = 1.15.) Values are means ± SEM. The results of the one-way analysis of variance are displayed. * *p* < 0.05, ** *p* < 0.01, *** *p* < 0.001, and **** *p* < 0.0001 between groups.

Consistent with the systemic loss of body mass in the MSP and HU models, pronounced atrophy was observed in the muscles of the hindlimb of the unloaded groups. The wet masses of several key muscles were significantly reduced after the four-week unloading period compared with the NOR group. The most affected muscles included the slow-twitch dominant soleus, mixed-fiber gastrocnemius, and quadriceps. Furthermore, significant atrophy was recorded in the anterior compartment muscles, tibialis anterior (TA), and extensor digitorum longus (EDL) (Figure 3A). Histological analysis revealed that the loss of muscle mass was concomitant with significant atrophy at the cellular level (Figure 3B). Quantification of the soleus muscles revealed a substantial reduction in fiber cross-sectional area in both the unloaded groups compared with the ground-based NOR group. Beyond structural atrophy, unloading induced a profound alteration in muscle fiber-type phenotype. We observed a significant decrease in the endurance-optimized, slow-twitch type I fibers, coupled with an increase in fast-twitch type II fibers (Figure 3C–E). This fiber-type shift, from fatigue-resistant to being more fatigue-prone, indicates a fundamental change in the muscle’s metabolic and contractile characteristics, aligning with a reduced endurance capacity. Additionally, musculoskeletal force generation was measured using the four-limb grip strength test. No significant decrease was observed in grip strength during the initial two-week interval. However, by the fourth week, a pronounced and significant decline in force was noted in the MSP and HU unloaded cohorts when compared with the NOR group (Figure 3F). Collectively, these results demonstrate that simulated microgravity not only results in a phenotypic shift in muscle fiber but also causes a decrease in contractile force in the MSP and HU unloaded models. This remodeling and impairment ultimately make muscle mechanically weak and susceptible to dysfunction.

### 2.4. RNA Sequencing Reveals Inflammation-Induced Apoptosis in MSP and HU

**Figure 4:**
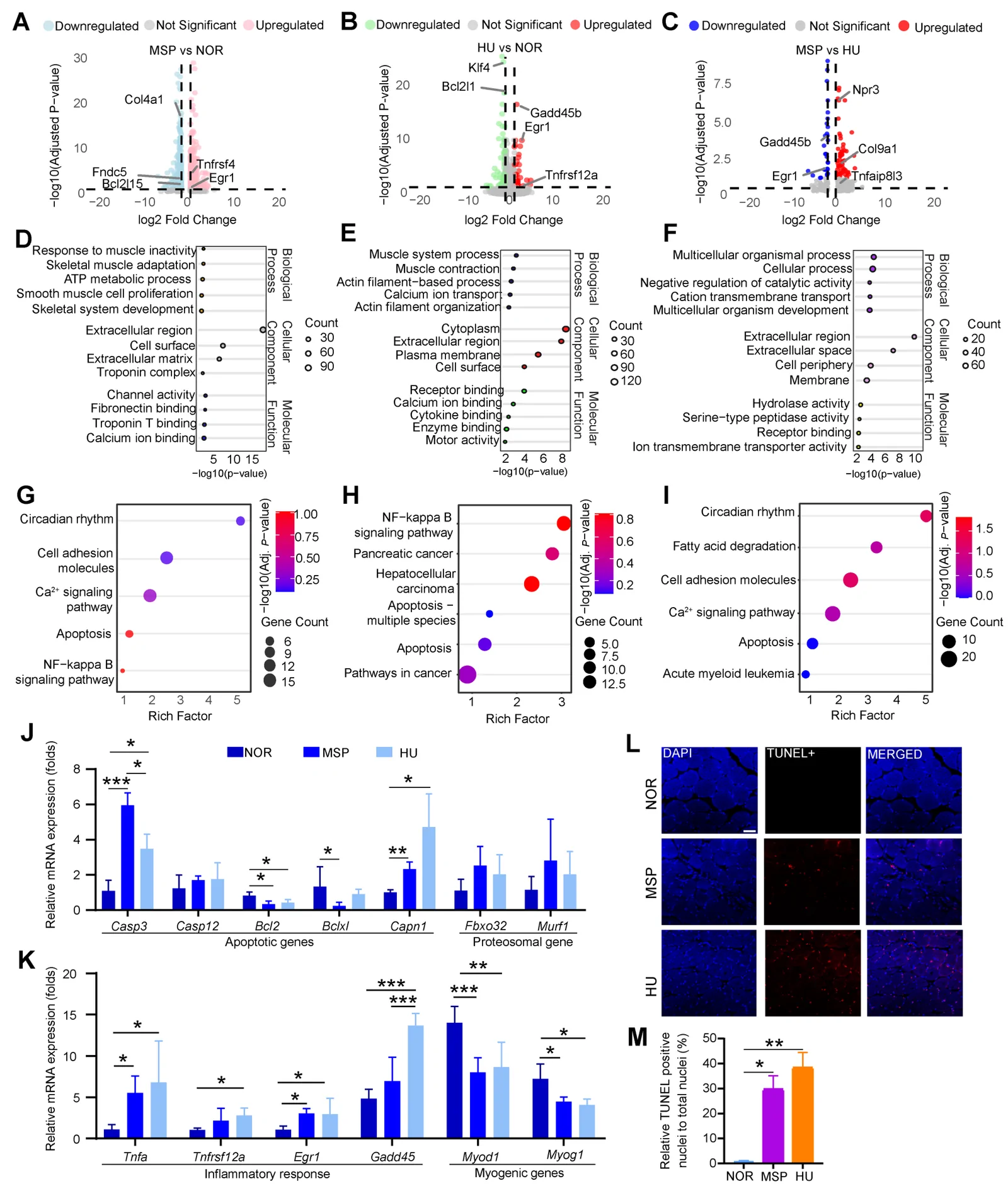
RNA sequencing revealed upregulation of inflammatory and apoptotic genes in the unloading cohort. (**A**–**C**) Volcano plots displaying differentially expressed genes in the NOR, MSP, and HU groups, depicting significant upregulated and downregulated genes involved in muscle atrophy during 4 weeks of disuse. (**D**–**F**) Significant biological, cellular, and molecular processes were downregulated in GO analysis between the NOR and MSP, NOR and HU, and MSP and HU groups, respectively. (**G**–**I**) Kyoto Encyclopedia of Genes and Genomes (KEGG) analysis between the NOR and MSP, NOR and HU, and MSP and HU group, respectively. (**J**,**K**) qPCR validation of apoptotic, inflammatory, ubiquitin ligase, and myogenic genes among three different groups (*n* = 3–5) (Cohen’s d: *Casp3*—NOR vs. MSP = −8.09, NOR vs. HU = 2.90, MSP vs. HU = 3.27; *Bcl2*—NOR vs. HU = 2.39, *Bclxl*—NOR vs. MSP = 3.00; *Capn1*—NOR vs. MSP = −4.26, NOR vs. HU = −2.90; *Tnfa*—NOR vs. MSP = −2.91, NOR vs. HU = −2.70; *Tnfrsf12a*—NOR vs. HU = −2.78; *Egr1*—NOR vs. MSP = −3.81, NOR vs. HU = −2.9; *Gadd45*—NOR vs. HU = −6.02, MSP vs. HU = −2.44; *Myod1*—NOR vs. MSP = 2.84, NOR vs. HU = 1.74; *Myog1*—NOR vs. MSP = 1.69, NOR vs. HU = 1.62). (**L**,**M**) IF TUNEL assay and quantification of TUNEL-positive nuclei in different groups (*n* = 4). Scale bar: 50 µm. (Cohen’s d: NOR vs. MSP = −3.78, NOR vs. HU = −4.23.) Values are means ± SEM. The results of the one-way analysis of variance are displayed. * *p* < 0.05, ** *p* < 0.01, and *** *p* < 0.001 between groups.

Differential gene expression of the MSP and HU groups when compared with the NOR group revealed that both unloading paradigms induce significant and similar transcriptional changes in terms of muscle atrophy (Figure 4A–C). Principal Component Analysis (PCA) demonstrated a close alignment between the HU and MSP cohorts, indicating a shared genomic response to mechanical unloading that is distinct from the NOR group. Analysis of the top differentially expressed genes, ranked by adjusted *p*-value, identified several key players potentially driving muscle atrophy. Among the genes, there is a significant downregulation of pro-survival members of the *Bcl-2* superfamily, indicating that apoptotic signaling may be a contributing mechanism to muscle loss. Furthermore, several genes associated with protein degradation were upregulated, including the E3 ubiquitin ligases *Fbxl13* and *Fbxl22*, implicating the ubiquitin-proteasome system in proteolysis. CXC motif chemokine ligand 1 (*Cxcl1*) was also elevated, suggesting the activation of an inflammatory response that can contribute to the atrophic environment. Concurrently, we observed that Early Growth Response 1 (*Egr1*), a transcription factor involved in the response to mechanical stress, significantly increased. This upregulation of *Egr1* suggests the recruitment of downstream pathways, including those involving the TNF family, in response to mechanical unloading. Reports have suggested that unloading increases *Egr1* expression in the tendon and muscle [16]. Another transcription factor, *Klf4*, which regulates cell differentiation, development, and inflammatory signaling via NF-κB, was also differentially upregulated. Its altered expression points to a potential dual role in coordinating the cell cycle arrest and pro-inflammatory signals that characterize atrophying muscle. The myogenic genes *Myog* and *Myod* were also downregulated and confirmed through qPCR.

Gene ontology (GO) downstream analysis demonstrated “muscle inactivity” in the MSP group and a significant decrease in biological processes related to “actin-based movement” and “muscle contraction” reflecting a state of mechanical stress in the HU unloaded groups. Cellular processes also indicate a decrease in the troponin complex, which may support the idea of decreased calcium binding and lowered muscle force (Figure 4D–F). Crucially, systematic pathway analysis using the KEGG resource confirmed significant upregulation of the apoptosis and NF-κB signaling pathway itself. It was reflected in the upregulation of pro-inflammatory cytokines such as the TNF superfamily, which can perpetuate the inflammatory environment and directly activate apoptotic signaling (Figure 4G–I). The apoptosis and NF-κB signaling pathway is a well-characterized regulator of muscle mass, particularly in disuse conditions such as unloading and immobilization, where it coordinates inflammatory and apoptotic processes to drive atrophy [17]. Our transcriptomic data provides compelling evidence that apoptosis is central to the atrophy observed in the MSP and HU models. The convergence of our GO and KEGG results positions the apoptosis and NF-κB pathway as a master regulator integrating the inflammatory and apoptotic signals that ultimately lead to muscle loss in our ground-based microgravity simulation models, and further validation was performed through qPCR and an immunofluorescence TUNEL assay of apoptosis (Table 1) (Figure 4J–M).

### 2.5. Overall Bone Health Is Compromised in the MSP and HU Groups and Poses a Risk of Disuse Osteoporosis

**Figure 5:**
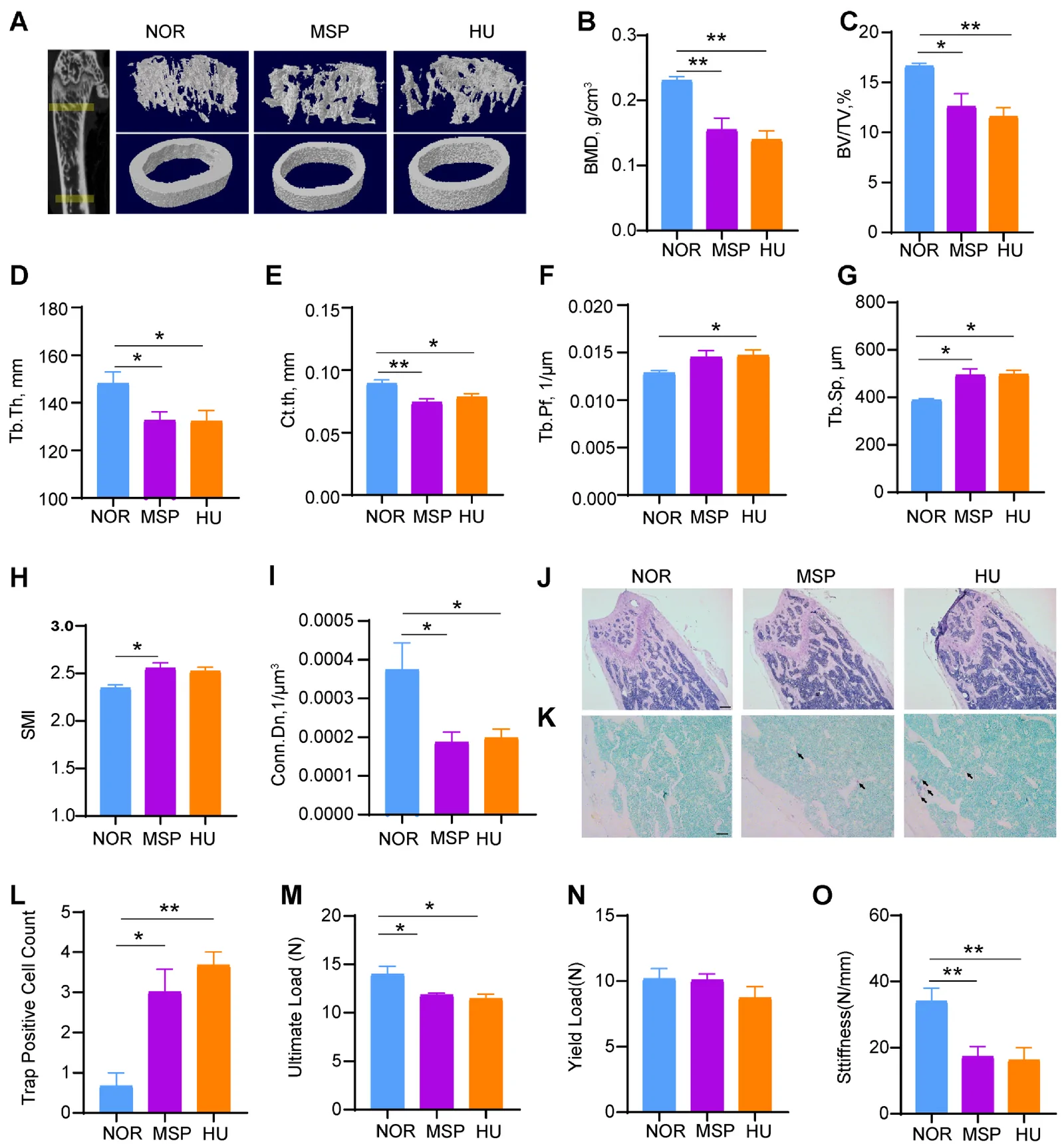
Unloading induces trabecular bone loss, enhanced osteoclastogenesis, and reduced femoral strength, collectively increasing susceptibility to disuse osteoporosis. (**A**) Quantitative micro-computed tomography pictures of trabecular (upper panel) and cortical (lower panel) sections of the NOR, MSP, and HU groups. (**B**–**I**) Quantitative analysis of BMD (bone mineral density) (Cohen’s d: NOR vs. MSP = 1.76, NOR vs. HU = 2.57); BV/TV (bone volume/total volume) (Cohen’s d: NOR vs. MSP = 1.25, NOR vs. HU = 2.29); Tb.Th (trabecular thickness) (Cohen’s d: NOR vs. MSP = 1.90, NOR vs. HU = 1.32); Ct.th (cortical thickness); Tb.Pf (trabecular perforation) (Cohen’s d: NOR vs. HU = −1.09); Tb.Sp (trabecular separation) (Cohen’s d: NOR vs. MSP = −1.09, NOR vs. HU = −1.48); SMI (structural model index) (Cohen’s d: NOR vs. MSP = −1.32); and Conn.Dn (connectivity density) for the NOR, MSP, and HU groups, respectively (*n* = 9–12) (Cohen’s d: NOR vs. MSP = 1.15, NOR vs. HU = 1.12). (**J**–**L**) HE and TRAP staining of osteoclasts (black arrow indicates TRAP-positive cells), and quantitative analysis of positive cells (*n* = 3). Scale bar: 100 µm and 50 µm, respectively. (Cohen’s d: NOR vs. MSP = −2.86, NOR vs. HU = −5.20.) (**M**–**O**) Three-point bending test using the tibial bone of the NOR, MSP, and HU groups, respectively (*n* =8–12). Ultimate load (Cohen’s d: NOR vs. MSP = 1.29, NOR vs. HU = 1.28). Stiffness (Cohen’s d: NOR vs. MSP = 1.42, NOR vs. HU = 1.24). Values are means ± SEM. The results of the one-way analysis of variance are displayed. * *p* < 0.05 and ** *p* < 0.01 between groups.

Micro-computed tomography (µCT) analysis revealed that the MSP and HU groups suffered significant skeletal deterioration when compared with the normal weight-bearing (NOR) group (Figure 5A). These results depict a parallel phenotype of disuse osteoporosis. The MSP and HU groups revealed a profound attenuation in overall bone mineral density (BMD) and bone volume fracture (BV/TV), and this reduction is apparent in the cortical and trabecular compartments, as indicated by decreases in cortical (Ct.Th) and trabecular (Tb.Th) thicknesses (Figure 5B–E). A rise in trabecular perforation (Tb.Pf), trabecular separation (Tb.sp), and structure model index (SMI) exhibited more fragile structural integrity of the unloaded bones, and a deficit in connectivity density (Conn.D) of the trabecular interconnection exhibited severe disconnection (Figure 5F–I). This analysis revealed the topologic disintegration of the different bone networks. To validate the bone resorption, tartrate-resistant acid phosphatase (TRAP) staining to visualize osteoclast activity was assessed and resulted in increased TRAP-positive osteoclast activity in the MSP and HU models when compared with the non-loaded group (Figure 5J–L). We further analyzed the load-bearing capacity of the bones using the tibial long bone for the three-point bending test and observed a striking reduction in bone mechanical properties. Maximum load and stiffness were significantly hampered in the MSP and HU groups when compared with the NOR group. This demonstrates that unloading not only affects bone mass but also critically diminishes the skeletal potency and increases the chances of fractures (Figure 5M–O).

### 2.6. Bone Proteomics Reveal Platelets and Complement Cascade Activation in the MSP and HU Groups

**Figure 6:**
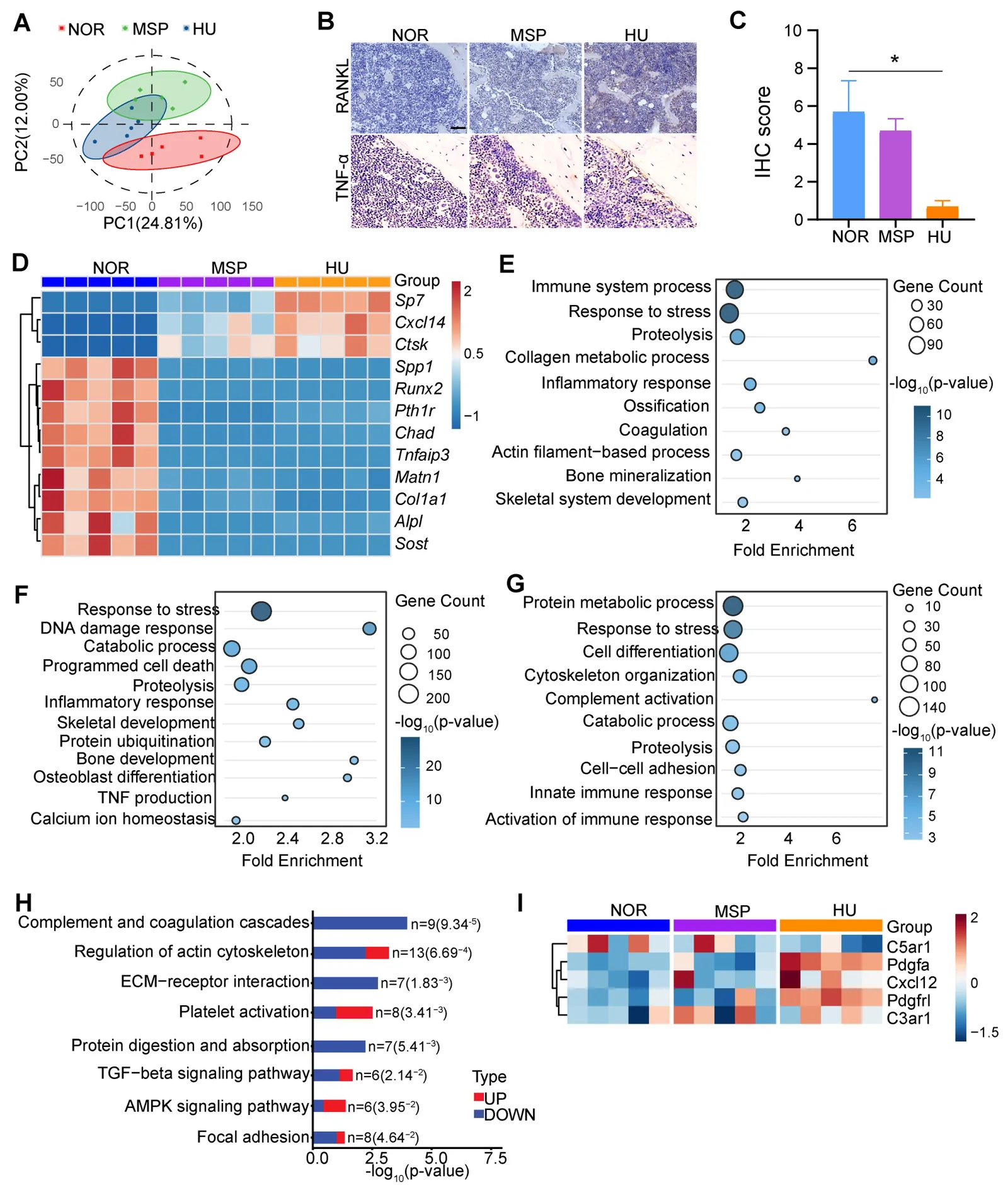
Bone proteomics reveals activation of platelet signaling and complement pathways in unloaded groups. (**A**) PCA component of the three groups. (**B**,**C**) Immunohistochemistry analysis of RANKL and TNF-α (*n* = 3). Scale bar 50 µm. (Cohen’s d: NOR vs. HU = 2.40). (**D**) Heatmap illustrating differentially expressed proteins in the three models. (**E**,**F**) Gene ontology (GO) analysis of MSP vs. NOR and HU vs. NOR, respectively. (**G**,**H**) Kegg and GO of signaling pathways affecting the MSP and HU groups. (**I**) Heatmap of genes involved in the complement system. Values are means ± SEM. The results of the one-way analysis of variance are displayed. * *p* < 0.05 between groups.

Microarchitectural quality and strength were severely degraded in the MSP and HU groups, thus characterizing disuse osteoporosis. This reorganization was driven by the upregulation of osteoclastogenic genes involved in bone resorption. Proteomic profiling elucidated the molecular genes governing osteoclast differentiation and activation. Principal Component Analysis (PCA) reveals distinct segregation of genes between the unloaded groups and the NOR group, whereas minimum variance observed within the unloaded cohorts underscored substantial remodeling of the bone landscape during mechanical unloading (Figure 6A). Inside this modified molecular signature, we identified Cathepsin K as a crucial downstream effector of RANKL signaling that is necessary for the breakdown of the bone matrix [18]. In the MSP and HU models, Cathepsin K expression was significantly increased, offering direct functional proof of increased osteoclast activity and bone resorption. Apart from the canonical RANKL pathway, inflammatory genes were also upregulated in the unloaded conditions. Notably, enhanced expression of TNfaip3, a potent regulator of NF-κB signaling, illustrates the activation of TNFα-mediated inflammatory response and contributed to osteoclast activation in the unloaded models (Figure 6B). The expressions of RANKL and TNFα were confirmed with immunohistochemistry analysis (IHC) (Figure 6B–D).

**Figure 7:**
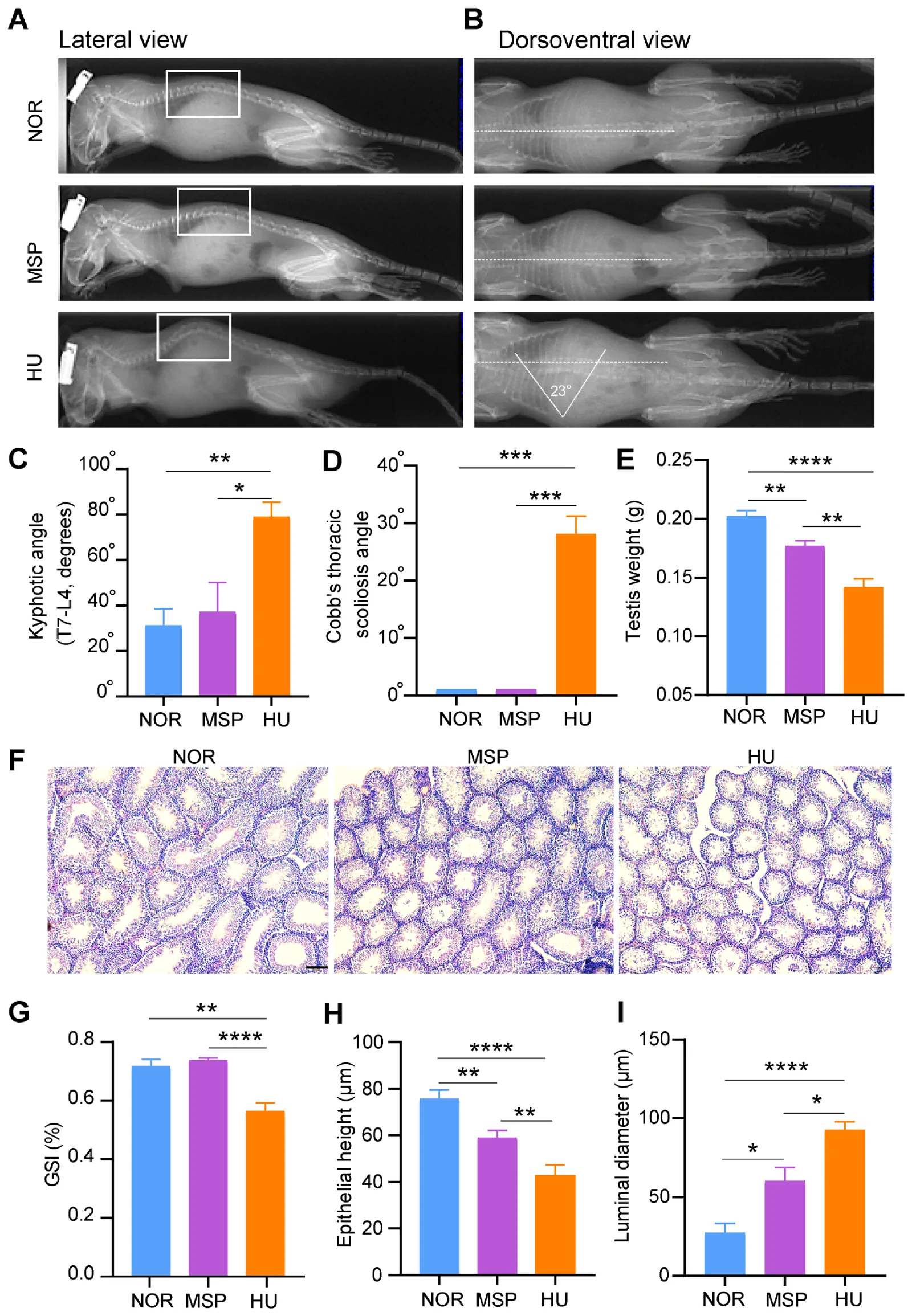
MSP induces musculoskeletal atrophy and reproductive dysfunction without spinal kyphosis. (**A**,**B**) Lateral and dorsoventral sides of the whole spinal cord, focusing on the thoracic segments of the NOR, MSP, and HU groups, respectively (*n* = 3). The box is drawn to highlight the spinal curvature in three cohorts, and the straight lines indicate the angle shift in the HU model. (**C**,**D**) Graphical comparison of kyphosis (Cohen’s d: NOR vs. HU = −3.92, MSP vs. HU = −3.21) and scoliosis (Cohen’s d: NOR vs. HU = −6.85, MSP vs. HU = 6.85) angles in the three groups, respectively (*n* = 3). (**E**) Testis weight of the NOR (*n* = 9), MSP (*n* = 12), and HU (*n* = 10) models in grams. (Cohen’s d: NOR vs. MSP = 1.63, NOR vs. HU = 2.98, MSP vs. HU = 1.73) (**F**) Representative HE images of the NOR, MSP, and HU cohorts. Scale bar: 100 µm. (**G**) The GSI (gonadosomatic index) is a measure of relative gonad mass, calculated as 100 times the gonad weight divided by the total body weight (Cohen’s d: NOR vs. HU = 1.77, MSP vs. HU = 2.41.) (**H**,**I**) The quantification analysis of epithelial height (Cohen’s d: NOR vs. MSP = 1.52, NOR vs. HU = 2.84, MSP vs. HU = 1.39) and luminal space (Cohen’s d: NOR vs. MSP = −2.15, NOR vs. HU = −4.83, MSP vs. HU = −2.20) of the three groups, respectively (*n* = 5–12). Values are means ± SEM. The results of the one-way analysis of variance are displayed. * *p* < 0.05, ** *p* < 0.01, *** *p* < 0.001, and **** *p* < 0.0001 between groups.

A significant disruption of biological pathways in response to mechanical unloading was identified by global transcriptome profiling. When comparing the MSP and HU cohorts to the NOR group, GO enrichment analysis revealed a significant upregulation of pathways related to cellular response to stress, programmed cell death (apoptosis), and inflammatory response (Figure 6E–G). To identify the specific upregulated genes of this inflammation, we performed a KEGG pathway analysis, allowing us to identify significant activation of the complement system and platelet activation pathways in the MSP and HU cohorts (Figure 7H). Notably, we detected model-specific divergence, where platelet activation was more prominent in the MSP group, whereas complement cascade activation was more pronounced in the HU group (Figure 6H).

Apart from TNFα directly stimulating osteoclast activation, we also noted platelet activation and the consequent release of cytokines, growth factors, and chemokines, including PDGFR, PDGF, and CXCL12 (Figure 6I) in the unloaded groups. Interactions between platelets and endothelial cells, macrophages, and immunological populations exacerbate inflammatory responses, perpetuate chronic bone resorption, and establish a pro-inflammatory and osteoclastogenic microenvironment [19,20]. PDGF binds to specific tyrosine kinase receptors and activates intracellular signaling pathways, including the PI3K/AKT and MAPK pathways, and enhanced stimulation of these pathways further increased spontaneous osteoclastogenesis by maintaining constant RANKL production. Our transcriptomic findings provide a molecular basis for further investigation into the role of platelet-derived signaling in unloading-induced bone loss, although direct evidence at the protein level remains to be confirmed.

Additionally, we observed increased regulatory activity of the complement system (Figure 6I). The cleaved products C3a and C5a modulated osteoblast differentiation and triggered the release of cytokines such as IL-6 and RANKL, thereby regulating the bone microenvironment and balancing osteogenesis and osteoclast activity [21,22].

### 2.7. MSP Mice Exhibit Musculoskeletal and Reproductive Deficits Without Spinal Deformity, Overcoming a Limitation of the HU Model

Beyond the muscle and bone phenotypes described above, we observed that unloading induced additional abnormalities in the axial skeleton and male reproductive system. Previous reports revealed that prolonged tail hanging induced severe spine deformity and caused kyphosis in rats [23]. Ethics emphasizes subject welfare and authorizes the selection of experimental models that promote humane study by minimizing stress. In the HU model, tail unloading induces biomechanical stress on the spinal column, resulting in adverse damage to the spine (Figure 7A). This force concentration causes pathological spinal curvature and thoracic kyphosis, often accompanied by scoliosis, which is a lateral deviation indicating asymmetric loading (Figure 7B). In contrast, the MSP model, utilizing whole-body unloading, demonstrates superior results. Radiographic analysis reveals that the MSP group maintained stable, normal spinal alignment, accounting for a more uniform distribution of gravitational counterforces across the torso (Figure 7C,D). Consequently, the MSP model provides a more physiologically appropriate and ethically docile platform for simulating microgravity, as it significantly reduces stress-induced morphological artifacts.

Reproductive organs show vulnerability to damage due to reduced gravity [24]. Quantitative analysis reveals that testis mass decreased in the MSP model relative to that in the NOR cohort, whereas a higher significant decrease was observed in the HU model when compared with the NOR group (Figure 7E). However, the MSP group showed a critical phenotypic dissimilarity from the HU group: maintained testis integrity (Figure 7F). Moreover, histomorphometry evaluation of the seminiferous epithelium revealed a significant increase in luminal space and a reduction in epithelial diameter in testes in the HU group, morphological indicators of germ cell depletion, and compromised spermatogenesis changes that were markedly greater in the HU than the MSP model (Figure 7G–I).

This disparity suggests that whereas both models replicate musculoskeletal disuse, the HU model precipitates a significant secondary hypogonadal condition, probably caused by systemic stress and disturbance of the hypothalamic–pituitary axis. The lack of off-target endocrine pathologies in the MSP model indicates that it offers a more precise simulation of microgravity, isolating the effects of mechanical unloading from the confusing consequences of significant neuroendocrine stress. Thus, the MSP may be more effective for examining the direct skeletal and muscular adaptations to disuse, facilitating clearer mechanistic understanding of spaceflight-related atrophy.

## 3. Discussion

The principle findings of this comparative study are as follows: (1) The novel MSP apparatus proved to be an advanced platform for studying simulated microgravity effects at the ground level, as it exhibited less stress and systemic inflammation in mice compared with the aggravated inflammatory response in the HU group. (2) The MSP apparatus provides better laboratory animal welfare and yields consistent physiological data, for example, natural spinal alignment, as well as controlled housing environments from the thermal heating system, thus providing a better apparatus for study. The MSP apparatus embodies the principles of the 3Rs, particularly Refinement, by significantly reducing pain, distress, and systemic inflammation in the experimental animals, thereby promoting improved welfare and more reliable experimental outcomes. (3) The MSP’s central axis rotation system offers the potential for future hypergravity studies, which remain to be experimentally validated. This dual-function mechanism and robust analog for studying exercise-related paradigms and the better housing environment for the animals made it possible to extend this apparatus for cognition and learning studies under simulated microgravity conditions.

Under normal physiological conditions, bone remodeling preserves homeostasis through bone-forming osteoblasts and bone-resorbing osteoclasts. However, prolonged exposure to spaceflight disrupts this balance and causes abnormal mechanical stimulation of the bone, leading to a decrease in bone mineral density, increased risk of fractures, and premature osteoporosis [25,26]. Skeletal muscle similarly adapts to unloading, with cosmonauts experiencing a 20% average reduction in muscle mass and nearly 30% decrease in strength after one month of microgravity [27]. Altogether, the diminished gravity environment leads to a decrease in skeletal muscle mass and impedes space travel by increasing the risk of injury when returning to Earth or encountering hypergravity conditions such as on Mars. To mitigate these risks and develop effective countermeasures, elucidating the cellular and molecular mechanisms driving low-gravity effects on skeletal muscle is essential. Our specialized apparatus provides a platform for investigating these degenerative processes at the molecular and cellular levels in small animals such as mice, rats, and even rabbits. By enabling precise, reproducible analysis of musculoskeletal tissues under simulated microgravity conditions, our equipment facilitates the mechanistic studies needed to develop effective countermeasures.

Increased systemic inflammation is a crucial signal in metabolic studies. In the acute period, we observed severe inflammation in hindlimb unloading via tail hanging, with prolonged application restricting blood circulation and causing pronounced inflammation, tissue damage, and necrosis. Surgical tail methods also resulted in severe skin erosion and bleeding requiring extended recovery, compromising experimental reliability [14]. Such outcomes affected the reliability of the experimental results. Herein, we observed enhanced IL1β and IL6 infiltration in the HU cohort, less inflammatory response, and more relaxed animal conditions in the MSP apparatus. Ambient temperature affects net energy balance and metabolic outcomes [28]. It has been reported that housing mice at 22 °C caused premature cancellous bone loss, whereas thermoneutral conditions (24–26 °C) prevented such a condition [29,30]. Thus, cold-induced stress plays a crucial confounding factor in animal behavior and metabolic studies. The MSP’s thermal heating system maintains 26 °C throughout the experiment, overcoming this confounding factor. Another cofactor is severe stretch in the tail in the HU group. Studies report that hindlimb unloading in rats caused a mild prediabetic state, with signs of pressure hyperalgesia and lumbosacral skeleton stretch [23]. In this study, we also observed higher vertebral stretch in the HU cohort that resulted in kyphosis and scoliosis-like deformities, whereas the MSP cohort did not suffer from any such deformities, establishing it as a superior platform for reliable microgravity research.

Bone and skeletal muscle tissues exhibit high responsiveness to mechanical loading conditions. Our study depicted severe muscle atrophy, indicated by diminished grip strength and decreased soleus cross-sectional area. Mechanistically, transcriptomic profiling and qPCR revealed apoptosis as the major pathway driving unloading-induced muscle atrophy in the MSP and HU models. While our transcriptomic data provides a strong foundation for hypothesis generation, functional experiments such as pathway inhibition and rescue studies are needed to establish causal links. Micro-CT analysis revealed severe disuse osteoporosis in both groups, characterized by significant bone loss and structural weakening driven by markedly increased osteoclast activity. This study focuses on diverse cell types comprising musculoskeletal tissues and their intercellular interactions while presenting potential countermeasures to mitigate microgravity-induced bone and muscle changes. Currently, the impact of reduced gravity on the hypothalamic–pituitary–gonadal (HPG) axis in female and male astronauts and its effect on reproductive functionality remains poorly understood [31,32]. Previous reports indicate that 7-day suspension decreased testosterone secretion in male mice, disrupting testicular morphology and molecular phenotypes [24]. Our study found similar findings, with testis morphology disrupted in both models, though more severe in the HU model, quantified by decreased testis weight, reduced epithelial height, and increased seminiferous tubule luminal diameter. The greater severity in the HU group likely reflects pronounced mechanical stretch in the caudal area, whereas the MSP jacket stabilized the pelvic region, reducing structural damage.

## 4. Technological Advancement and Future Development

For the first time, we designed a customized jacket made of soft nylon, providing comfort and ease. We are actively developing an advanced version of the jacket with built-in sensors to detect cardiopulmonary signals. This proposed model not only mimics ground-based microgravity but also delivers less distress to the mice, serving as an innovative approach for exploring future research directions and addressing knowledge gaps as space exploration advances. Additionally, the MSP apparatus incorporates a larger animal housing capacity, accommodating more mice per experiment while maintaining individual compartments with separate food and water supplies for each animal. This design facilitates precise monitoring of individual health and experimental outcomes, as food intake and water consumption can be tracked per mouse. Furthermore, the system is equipped with an integrated thermal control mechanism to maintain consistent temperature throughout the experiment, eliminating cold-induced stress as a confounding variable. The individual housing design also enables easy and efficient collection of feces and other biological samples from each mouse separately, allowing for detailed metabolic and gut microbiome analyses on a per-animal basis.

## 5. Study Limitations

Moreover, for the first time, we introduced an ergonomically advanced machine with the capability to rotate at different speeds to mimic hypergravity experienced during launch or to be used as a countermeasure to deal with adverse effects of microgravity. However, hypergravity studies were not performed in this study. We also acknowledge that maintaining artificial gravity in space is a critical challenge for future space travel. Current countermeasures against microgravity-induced deconditioning such as bone loss and muscle atrophy rely primarily on exercise protocols, which have proven insufficient for long-duration missions [33,34]. Research increasingly focuses on artificial gravity technologies, with centrifugation being the most promising approach, achievable via large rotating habitats or short-radius human centrifuges [35,36]. Recent advancements in high-expansion-ratio deployable structures (HERDS) tested in microgravity demonstrate potential for kilometer-scale rotating habitats for long-term space travel [37]. These developments underscore the importance of ground-based analogs such as MSP for informing and validating countermeasure strategies. Although we have extended validation to include female mice over an eight-week unloading protocol, further investigations comparing MSP findings with real spaceflight data and across different age groups are needed to establish general applicability [38,39]. Thus, whether these findings extend to females remains unknown. Additionally, recovery from microgravity-induced deconditioning upon return to Earth addressed through cardiovascular and resistance exercise rehabilitation, as well as lower body negative pressure (LBNP) to restore orthostatic tolerance, remains an important research area [40,41]. Our MSP apparatus, providing a more reliable and less stressful model, may serve as a valuable platform for testing recovery strategies through future hypergravity studies.

## 6. Methods

### 6.1. Animals

Ten-week-old male C57 mice were purchased from Guangdong Medical Laboratory Animal Center (Foshan, China) and housed in SPF conditions in a room controlled for temperature (20–26 °C) and humidity (40–70%), with lights on from 8:00 AM to 8:00 PM. After one week of adaptation, the mice were randomly divided into three groups: MSP (12 n), HU (12 n), and NOR (9 n). All were fed a normal chow diet. Mice in the MSP group were anesthetized using isoflurane, wrapped in a jacket, and hung on metal chains in the apparatus, whereas mice in the HU group had their tails wrapped with nylon cable ties and surgical tape and were hung through a metal wire. To simulate microgravity in the MSP and HU groups, the head was tilted toward the ground at around 30° to create an angle for cephalic fluid shift. Mice in the NOR group were housed in a normal bedding cage.

### 6.2. Tissue Collection

The mice were humanely euthanized at 4 weeks of unloading. Then, the lower limbs were carefully prepared by excising the skin, collecting different muscles, and then dissecting connective tissues away from the femur and tibia. The limbs were sectioned at the ankle and hip joints to isolate clean bone samples. The left pair of muscles was snap-frozen for RNA sequencing, and the right pair was used for quantification. Similarly, the right tibia was snap-frozen to preserve the integrity of the RNA for subsequent sequencing analysis. The right femur was frozen until mechanical testing. The left femur and tibia were fixed in 4% paraformaldehyde (AR1068, BOSTER, Wuhan, China) for 72 h and then transferred to a 70% ethanol solution for micro-CT scanning. Following micro-CT, the femur was prepared for plastic embedding, allowing for mineral examination, while the tibia was decalcified and embedded in paraffin for further histological analysis.

### 6.3. Immunostaining and Image Analysis

For muscle tissue, after dewaxing and rehydrating the paraffin sections, the heat antigen retrieval step was performed with a citrate-based antigen retrieval solution (PH 6.0) (G1202, Servicebio, Wuhan, China), then treated with a tissue spontaneous fluorescence quencher (G1221, Servicebio), and permeabilized with PBS containing 0.4% Triton X-100 for 30 min at room temperature. After washing with the PBS buffer, the tissues were incubated with 3% BSA for 30 min. Then, overnight incubation was performed with the first primary antibody anti-mouse slow MYH7 antibody (GB112131, Servicebio, dilution 1:500), and incubation with the HRP-conjugated secondary antibody was performed the next day at room temperature for 1 h. After washing, the IF488-Tyramide (tsa) (G1231, Servicebio, dilution 1:800) dye was added for 10 min at room temperature, followed by a decolorizing wash. Subsequently, the heat antigen retrieval step was performed once again with the same primary antibody anti-mouse fast MYH1 antibody (GB112130, Servicebio, dilution 1:500), HRP-conjugated secondary antibody, and CY3-Tyramide (tsa) (G1223, Servicebio, dilution 1:800) dye used in the previous steps. Then, the tissues were counterstained with DAPI nucleic acid stain (Servicebio, G1012, dilution 1:1000). MYH7 (slow-twitch) and MYH1 (fast-twitch) myosin heavy chain isoforms were selected for immunostaining because mechanical unloading is known to induce a shift in fiber type composition, with preferential atrophy of fast-twitch fibers under microgravity conditions. DAPI was used as a nuclear counterstain to facilitate accurate fiber segmentation and morphometric analysis. Immunofluorescence images were scanned by Panoramic MIDI 3DHISTECT (Budapest, Hungary).

For morphometric analysis, muscle cross-sectional area (CSA) and fiber size distribution were quantified using ImageJ software (Fiji distribution, version 1.54n) with the MuscleJ plugin, enabling automated fiber segmentation based on immunofluorescence channels. The DAPI channel was used to identify nuclei, while the MYH7 (slow) and MYH1 (fast) channels classified fiber types. Fibers touching the image border or with irregular morphology were excluded. A minimum of three randomly selected fields per section and at least three sections per animal were analyzed. Mean CSA and fiber size distribution (100 µm^2^ bin width) were calculated for slow (Type I), fast (Type II), and total myofibers. All quantifications were performed in a blinded manner. The percentage of each fiber type was calculated as the number of fibers positive for the respective myosin heavy chain isoform divided by the total number of fibers counted per field.

For IHC, tibial sections (5 μm) from the paraffin-embedded samples were deparaffinized, the antigen was retrieved (G1202, Servicebio, China), and the sample was then blocked with 3% BSA for 30 min at room temperature. Overnight incubation at 4 °C with 52B83 RANKL (Proteintech, Rosemont, IL, USA, 241791B7) and TNFα (Santa Cruz, California, CA, USA, 52b83) was followed by treatment with HRP-labeled goat anti-mouse and anti-rabbit IgG. Slides were then hematoxylin-counterstained.

### 6.4. Histology

Testes were dissected, weighed, fixed in 4% paraformaldehyde for 24 h at 4 °C, embedded in paraffin, and sectioned at 5 µm thickness. Sections were stained with hematoxylin and eosin (H&E), and periodic acid-Schiff (PAS) for morphological evaluation. Histomorphometric analysis was performed on digitized images captured using a light microscope (Olympus CX31, Olympia, WA, USA) at 20× magnification. Seminiferous tubule diameter, epithelial height, and luminal diameter were measured in at least 50 round or nearly round tubules per testis, as previously described [42,43]. Testicular capsule thickness was measured at 10 randomly selected sites per section. All measurements were performed in a blinded manner.

### 6.5. TUNEL Assay

Apoptotic cells in tissue sections were detected using the One-step TUNEL cell apoptosis detection kit (Beyotime, Shanghai, China, C1090) following the manufacturer’s protocol with minor modifications. Briefly, paraffin-embedded tissue sections (5 µm thickness) were deparaffinized, rehydrated, and permeabilized with Proteinase K (20 µg/mL) for 30 min at 37 °C. After washing with PBS, sections were incubated with TdT reaction mixture (containing terminal deoxynucleotidyl transferase and fluorescein-labeled dUTP) for 1 h at 37 °C in a humidified chamber. Negative controls were prepared by omitting the TdT enzyme. Sections were counterstained with DAPI (1 µg/mL) to visualize all nuclei. TUNEL-positive cells (green fluorescence) and DAPI-positive nuclei (blue) were imaged using a confocal microscope (Zeiss LSM 880, Oberkochen, Germany) at 20× magnification. For each section, at least five randomly selected fields were captured, and the apoptotic index was calculated as the percentage of TUNEL-positive nuclei relative to total DAPI-positive nuclei. Quantification was performed using ImageJ software (Fiji distribution) with the Cell Counter plugin, and all analyses were conducted in a blinded manner.

### 6.6. Trap Staining

For osteoclast detection, femurs were fixed in 4% paraformaldehyde for 72 h, decalcified in 10% EDTA (pH 7.4) for 14 days, and embedded in paraffin. Sections (5 µm thickness) were deparaffinized and rehydrated through graded ethanol series. TRAP staining was performed using a commercial TRAP staining kit (Servicebio, G1050-50T). Briefly, TRAP working fluid was prepared using 50 μL of fuchsettia solution (G1050-2) and 50 μL of sodium nitrite solution (G1050-3) in a clean centrifuge tube to obtain a hexazo-azo fuchsin-red solution. Then, 100 μL of AS-BI phosphate substrate solution (G1050-4) was added to 100 μL of the hexazo-azo by-pursuing solution from step 1; the solution was pinched several times until thoroughly mixed. Subsequently, 1.8 mL of the reaction buffer (G1050-1) was drawn and added to the mixture from step 2, again mixing thoroughly. The mixed trap working solution was then left to stand for 20 min–1.5 h before filtering for use. After incubation of the sections in pure water at 37 °C for 2 h, we added the filtered TRAP solution dropwise, covered the tissue, and incubated the mixture at 37 °C in the dark for 20–30 min. Then, the solution was washed with water and the nuclei stained with hematoxylin staining solution. Lastly, the tissue slides were dehydrated until transparent and sealed with neutral resin. TRAP-positive osteoclasts were identified as multinucleated cells with wine-red/maroon cytoplasmic staining. For each specimen, at least five randomly selected fields per section were analyzed at 20× magnification using a light microscope (Olympus CX31). The osteoclast surface per bone surface (Oc.S/BS) and the number of osteoclasts per bone perimeter were quantified using ImageJ software (Fiji distribution) with the Cell Counter plugin. All quantifications were performed in a manner blinded to the experimental groups.

### 6.7. Serum Assay

Serum were obtained from the blood by centrifugation at 1000 rpm for 30 min and immediately stored at −80 °C. Serum measured using ELISAs were processed according to the manufacturer’s guidelines and measured against an inter-assay standard curve. Then, the measurement of biochemical markers of inflammation in the serum, including IL1β, TNFα, and IL6, were assessed according to the manufacturer protocols using the Mouse IL-1β ELISA Kit (PI301, Beyotime Biotechnology, Shanghai, China), MS Mouse TNF-α ELISA Kit (E-MSEL-M0002, Elabscience, Wuhan, China), and Mouse IL-6 ELISA Kit (E-MSEL-M0001, Elabscience). All samples were assayed in duplicate, and the optical density was measured at 450 nm using a microplate reader (Thermo Fisher Scientific, Waltham, MA, USA).

### 6.8. Micro-CT Scanning

The left femur and tibia were scanned using a high-resolution SkyScan 1176 micro-CT (Bruker, Bruges, Belgium) at a spatial resolution of 9 μm, using a source voltage of 60 kV and a source current of 416 μA, with 2 average frames at every 0.5 angle step, to assess the distalis region. Image reconstruction was performed using the NRecon software V1.6.9.8 (SkyScan, Kontich, Belgium), with a smoothing value set to 1, ring artifact reduction to 5%, and beam hardening correction to 40%. The grayscale threshold was controlled with a minimum of 80 Hounsfield units (HU) and a maximum of 255 HU. Subsequently, three-dimensional (3D) reconstructions of the region of interest (ROI) were generated using the CTvol software 2.3.2.0 (SkyScan, Kontich, Belgium). The ROI was specifically delineated above the distal metaphyseal growth plate. Finally, the BMD and micro-architectural parameters of the trabecular and cortical bone, such as bone volume fraction (BV/TV), trabecular thickness (Tb.Th), trabecular number (Tb.N), trabecular separation (Tb.Sp), and cortical thickness (Ct.Th) in the distal femur, were analyzed using the CTAn software v.1.20.8 (SkyScan, Kontich, Belgium).

### 6.9. Three-Point Bending Test

Right tibial samples were thawed from −80 °C and equilibrated in physiological saline at room temperature. The femurs were positioned in the grips of the testing machine (TSE503C, WANCE Testing Machine, Shenzhen, China), with the supports spaced 16 mm apart. The test parameters were set with a displacement limit of 9 mm and a load range of −500 N to 500 N, with positive values indicating axial tension. The machine was zeroed to ensure the femur was in an undeformed state. A force was applied at a rate of 5 mm/min, leading to progressive deformation until fracture occurred. Fracture was determined when the force dropped below 0.7 times the previous cycle’s value or to less than 0.5 times the maximum force. Continuous load and displacement data were recorded and stored for analysis.

### 6.10. Assessment of Grip Strength

The maximum grip strength was assessed using a grip strength meter with a connected mesh wire apparatus. Briefly, the mice were first positioned on the mesh platform, ensuring they gripped the grid with all four paws. Then, they were gently pulled horizontally by the base of their tail across the mesh wire until they released the mesh grid. This process was repeated for five trials, resting for 1 min between each trial, and the peak force was recorded. The average of the three highest peak force measurements was considered the overall grip strength. All grip strength assessments were conducted on the same day for all three experimental groups.

### 6.11. In Vivo Radiologic Imaging of Mouse Spine

To assess spinal deformities, mice were anesthetized via intraperitoneal injection of pentobarbital. Radiographs were obtained using a digital diagnostic X-ray machine. The mice were positioned in a standardized posture to ensure reproducibility—the snout, interorbital space, neck, and whole spine were aligned in a straight line, with limbs placed symmetrically to the trunk, avoiding any stretching of the body. Gingko CADx 2.4.1 software (Valladolid, Spain) was used to assess gross anatomical measurements and to measure the magnitude of the largest scoliotic and thoraco-lumbar kyphotic curves, according to the Cobb method [44].

### 6.12. Quantitative Real-Time PCR (qPCR)

Total mRNA was extracted using Trizol (15,596,018, Invitrogen, Carlsbad, CA, USA) reagent and reverse-transcribed with a reverse transcription PCR kit (RR036, Takara, Tahara, Japan). qPCR was conducted on a light cycler (Roche, Basel, Switzerland) using a SYBR quantitative real-time PCR kit (RR820, Takara, Japan). The expression of genes was amplified and detected using specific primer sets. Relative gene expression was determined using the ∆Ct method, with normalization to the endogenous control of the β-actin gene (Table 1).

### 6.13. RNA-Sequencing and Bioinformatics Analyses

The RNA was fragmented to about 300 bp in length using an ion fragmentation method. Using RNA as a template, cDNA first-strand synthesis was performed with a 6-base random primer and reverse transcriptase, followed by second-strand cDNA synthesis using the first-strand cDNA as a template.

After library construction, PCR amplification was used to enrich library fragments, followed by library size selection, resulting in a library size of 450 bp. Next, the library was quality-checked using an Agilent 2100 Bioanalyzer (Santa Clara, CA, USA), and total and effective library concentrations were measured. Then, according to the effective concentration of the library and the required sequencing data amount, libraries containing different index sequences (each sample was given a different index, and samples were distinguished based on the index in the sequencing data) were mixed in proportion. The mixed library was uniformly diluted to 2 nM, and single-stranded libraries were formed through alkaline denaturation. After RNA extraction, purification, and library construction, the samples underwent next-generation sequencing (NGS). Based on the Illumina sequencing platform, the libraries were sequenced using paired-end (PE) sequencing. Differential expression gene (DEG) analysis was conducted using DESeq2 (v1.4.5) with criteria set at *p*-value ≤ 0.05 or false discovery rate (FDR) ≤ 0.001. Gene ontology (GO) enrichment analysis and gene set enrichment analysis (GSEA) were performed, and the results were visualized with R packages. Significant terms were selected with a cutoff of *p*-value and/or FDR < 0.05.

For PCA, raw read counts were normalized using the DESeq2 variance-stabilizing transformation (VST) to stabilize variance across the expression range. PCA was performed on the top 500 most variable genes to reduce noise while retaining biologically relevant variation. Sample clustering was visualized using the prcomp () function in R (v4.3.0), and plots were generated using ggplot2 (v3.4.2). Replicates were assessed for tight clustering to confirm experimental reproducibility, and PC1/PC2 were annotated with the percentage of variance explained. Genes contributing most to each principal component were identified based on loading scores > |0.3| and used for downstream pathway interpretation. All analyses were performed on log_2_-transformed counts with a pseudo count of 1 to handle zero values.

For KEGG pathway enrichment analysis, differentially expressed genes (|log_2_FC| ≥ 1, adjusted *p* < 0.05) were submitted to clusterProfiler (v4.8.0) in R. Enrichment was performed using the enrichKEGG () function with the organism set to ‘mmu’ (Mus musculus). Significance was determined using Fisher’s exact test, and *p*-values were corrected for multiple testing using the Benjamini–Hochberg (BH) method to control the false discovery rate (FDR) at q < 0.05. Pathways with q < 0.05 and a minimum gene count of 2 were considered significantly enriched. The KEGG REST API (release 102.0) was accessed to obtain the most up-to-date pathway annotations. Enriched pathways were visualized as bar plots and dot plots using ggplot2, with the color gradient representing q-values and dot size representing gene count.

### 6.14. Statistical Analysis

All experiments were repeated thrice, and the results are expressed as mean  ±  SEM. Statistical analyses were conducted using GraphPad Prism 9.5. For comparisons between the three groups, data were analyzed using one-way ANOVA, followed by Tukey’s post hoc test for multiple pairwise comparisons. For direct comparisons between only two groups, unpaired Student’s *t*-tests were used. Statistical significance was defined as * *p* < 0.05, ** *p* < 0.01, *** *p* < 0.001, and **** *p* < 0.0001.

## Figures and Tables

**Table 1:** List of primers used for quantitative real-time PCR (qPCR).

Target Genes	Forward Primers	Reverse Primers
*Atrogin*	cagcttcgtgagcgacctc	ggcagtcgagaagtccagtc
*Murf1*	gtgtgaggtgcctacttgctc	gctcagtcttctgtccttgga
*Fbxo32*	cttctcgactgccatcctggat	tcttttgggcgatgccactcag
*Calpain*	ctgggcttcaaggaactgggt	gagccacccggacagacatc
*Myod*	cggcagaatggctacgacac	gcagtcgaggctcgacacag
*Myog*	cccatggtgcccagtgaa	gcagattgtgggcgtctgta
*Caspase3*	gatgtggacgcagccaacct	tcacacacacaaagctgctcc
*Caspase12*	cagatgaggaacgtgtgttgagc	ggaaccagtcttgcctaccttc
*Bcl2*	cctgtggatgactgagtacctg	agccaggagaaatcaaacagagg
*Bclxl*	gccacctatctgaatgaccacc	aggaaccagcggttgaagcgc
*Tnfa*	ggtgcctatgtctcagcctctt	gccatagaactgatgagagggag
*Tnfrsf12a*	gacctcgacaagtgcatggact	cgccaaaaccaggaccagacta
*Gadd45*	cctggaggaagtgctcagcaag	gtcgtcttcgtcagcagccag
*Egr1*	agcgaacaaccctatgagcacc	atgggaggcaaccgagtcgttt
*Actb*	atgctccccgggctgtat	aggcccagagcaagagagg

## Data Availability

All data in this study are presented in the manuscript. The source data that supports the findings of this study are available from the corresponding author upon request.

## Supplementary Materials

The following supporting information can be downloaded at: https://www.mdpi.com/article/10.3390/ijms27198806/s1.
