# Supplementary material for: Establishment and Validation of a Novel Microgravity Simulation Platform for Ground-Based Animal Experiments
Source: Int J Mol Sci. 2026 Oct 1;27(19):8806. doi: 10.3390/ijms27198806 (PMC13665397; doi:10.3390/ijms27198806)
Supplement: Supplementary file 1 [file ijms-27-08806-s001.zip › Supplementary figures. .pdf]

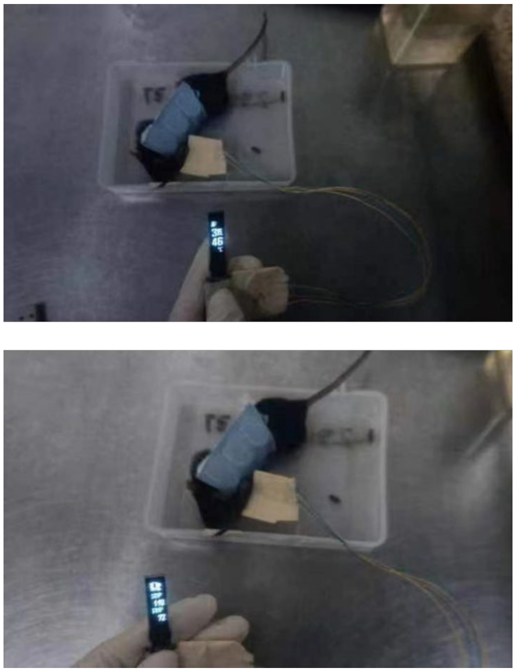

Figure S1- V1.0 of wearable measuring equipment.

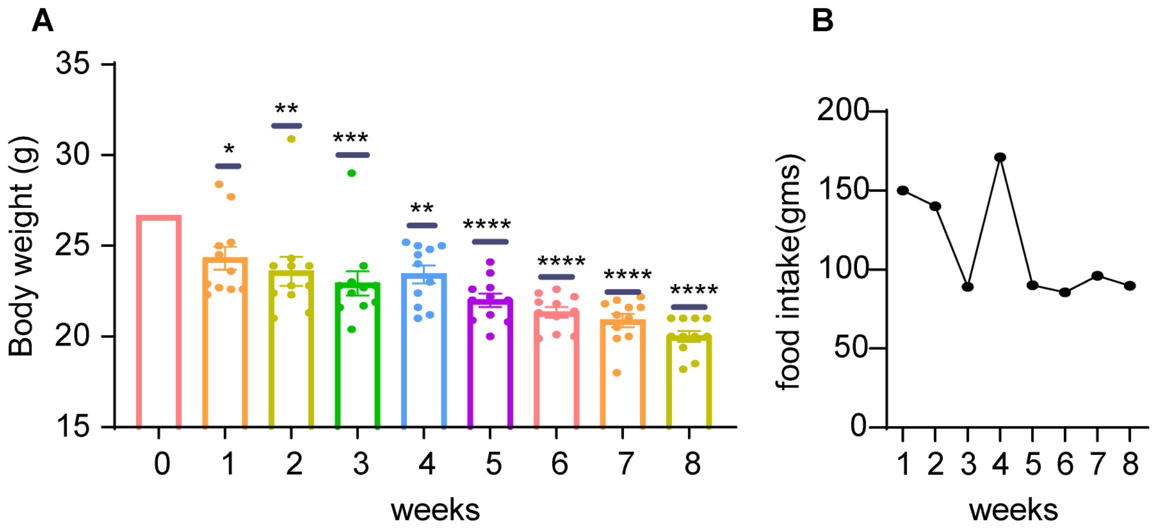

Figure S2- Body weight and food intake of female C57 mice during eight weeks of mechanical unloading. Data points represent mean ± SEM from independent experiments, with n = 12 mice. Statistical significance is denoted as *$p < 0.05$, **$p < 0.01$, ***$p < 0.001$, ****$p < 0.0001$ by Student's t-test.
